# Relationship between sleep quality and quality of life in patients with bipolar disorder

**DOI:** 10.5935/1984-0063.20190135

**Published:** 2020

**Authors:** Farshid Shamsaei, Samira Yadollahifar, Amir Sadeghi

**Affiliations:** 1 Behavioral Disorders and Substance Abuse Research Center, Hamadan University of Medical Sciences, Hamadan, Iran.; 2 Iran Department of Nursing, School of Nursing and Midwifery, Hamadan University of Medical Sciences, Hamadan, Iran.

**Keywords:** Sleep Quality, Quality of Life, Bipolar I Disorder

## Abstract

**Objective:**

Sleep disorder is one of the most classic symptoms of patients with bipolar I disorder (BID), which affects their quality of life (QOL). The current study aimed to determine the relationship between sleep quality and quality of life in patients with bipolar I.

**Methods:**

In this descriptive cross-sectional study, 180 patients with bipolar I disorder were selected using convenience sampling in Farshchian Psychiatric Center of Hamadan, Iran, in 2017. The data collection instruments were Pittsburgh Sleep Inventory and Brief Quality of Life Questionnaire. Data analysis was performed using Pearson’s correlation coefficient and stepwise multiple regression by SPSS 23.

**Results:**

The results indicated that 41.1% and 54.4% of patients with bipolar I disorder experienced low level of sleep quality and QOL, respectively. There was a statistically significant relationship between sleep quality and QOL (r=-0.571, *p*<0.001), so that low sleep quality has a negative impact on the QOL in these patients.

**Discussion:**

Patients with bipolar disorder suffer from sleep disorder affecting their QOL. Therefore, it is suggested that treatment and care interventions be designed and implemented to improve sleep quality and patients’ QOL. Moreover, treatment interventions of bipolar disorder are inseparable from the treatment of sleep disturbance.

## INTRODUCTION

Bipolar disorder is a severe and debilitating condition, and a general worldwide health problem existing in all world people, in any race or social class, with a global prevalence of 2.6-4.5%^1^.

Patients with bipolar I disorder experience periodical mania and depression, or may only have periods of mania. Symptoms appear in a variety of ways such as increased energy, increased self-confidence, high spirited thoughts, euthymia, increased irritability, and dangerous behaviors. One of the most common symptoms of bipolar I disorder, especially in the mania phase, is sleep disorder and reduced sleep. A sleep disorder can affect the disease treatment and recovery process, progression of the disease, and the QOL of these patients^2^^,^^3^.

Many patients with bipolar disorder suffer from other comorbid psychiatric disorders, such as anxiety and drug abuse, which have an adverse effect on their QOL and general health^3^^,^^4^. Sleep problems may vary at different stages of the disease. During mania, sleep disorders commonly manifest as reduced sleep, and according to many studies, sleep disorder varies from 69% to 99% in these patients^5^^-^7.

The irregular sleep pattern in patients with bipolar I disorder influences on the treatment and recovery process, and exacerbates the symptoms and recurrence of the disease. Diminished sleep quality and the sleep duration adversely affect various aspects of QOL including general health, physical-cognitive, and psychological function, resulting in the daily dysfunction and loss of individual performance^8^^,^^9^.

In the study of Fuente-Tomás et al.^10^, sleep disorder has been reported in over 60.5% of bipolar patients, which had negative effect on their QOL. In other words, sleep disorders significantly reduce QOL, as several studies suggested a significant relationship between sleep disorder and decreased QOL, which is also the case for bipolar patients^11^^,^^12^. Therefore, studying the relationship between these two variables will improve our knowledge of the problems of bipolar I patients, used in therapeutic and care interventions. In this regard, the current study aimed to determine the relationship between sleep disorders and QOL in bipolar patients.

## METHODS

This descriptive cross-sectional study was conducted in the second half of 2017 in Farshchian Psychiatric Center, Hamadan. The sample size included 180 patients with bipolar I disorder, which was calculated using G Power 3.1 software, according to the type of test (Pearson correlation) with a test power of 0.95, a significant level of 0.05, and the drop-out rate of 30%. Inclusion criteria were: adult patient, one-year history of illness, and diagnosis of bipolar I disorder based on DSM-5, as well as reading and writing literacy. On the other hand, the exclusion criteria included a diagnosis of comorbid psychiatric illness, history of drug abuse, chronic physical illness, mental disability and patients in the acute phase of the disease.

Sampling was performed in convenience method. The inpatients in psychiatric wards were selected based on the diagnosis of a psychiatrist after evaluating the medical records and the initial interview in case of meeting the inclusion criteria. Then, researchers explained the objective to ensure and communicate with the patients, and filled out the questionnaires in the self-report form, if they wished to participate in the research.

### Instruments

The data collection instruments included demographic questionnaire, Pittsburgh Sleep Quality Inventory, and Brief Quality of Life Questionnaire (QLQ). Pittsburgh Sleep Quality Inventory includes nine items to examine mental sleep quality, sleeping late, adequate sleep, sleep period, sleep disorder, sleeping pills and daily dysfunction. Each item has a score between zero and three (0 = normal status, 1 = weak and 2 = moderate and severe), totally ranging from 0 to 18. The higher the score, the lower the sleep quality will be, with scores above 6 indicating poor sleep quality^13^. A Persian version of questionnaire, validated in Iran was used in this study. The psychometric properties of the Persian version of the PSQI were acceptable^14^^,^^15^.

QLQ consists of 26 items with four subscales of physical health (7 items), mental health (6 items), social relationships (3 items) and environmental health (8 items). Two questions in the first item do not belong to any subscale and generally assess the health and QOL. The scoring was based on the Likert five-point scale (from one to five)^16^. The validity and reliability of the Persian version of this questionnaire had already been confirmed in Iran^17^.

### Data analysis

Data analysis was performed using SPSS 23. The statistical tests including frequency and mean were used to describe the data, while Pearson correlation coefficient and stepwise multiple regression were employed to examine the relationship between variables.

### Ethical considerations

This study was approved by the Ethics Committee of Hamadan University of Medical Sciences (IR.UMSHA.REC.1396.570). The written informed consents were obtained from research participants.

## RESULTS

The average age of patients was 28.4 years. Out of them, 55% were male, 45% female, and 36.7% had the secondary education (High School) diploma. Further, 62.3% were unemployed, 33.3% had duration of the illness equal or more than 49 months (Table 1).

**Table 1:** Percentage on the socio-demographic characteristics of caregivers.

Characteristics	N	%
**Sex**		
Male	99	55
Female	81	45
**Ages (years)**		
≥24	21	11.7
25 - 29	43	23.9
30 - 34	37	20.6
35 - 39	33	18.3
40 - 44	19	10.6
45 - 49	10	5.5
≤50	17	9.4
**Marital status**		
Single	81	45
Married	60	33.3
Divorced	24	13.4
Widowed	15	8.3
**Educational Level**		
Primary School	22	12.2
Secondary School	49	27.2
High School Diploma	66	36.7
University	43	23.9
**Employment status**		
Employed	17	9.4
Unemployed	112	62.3
Retired	6	3.3
Business	20	11.2
Agricultural worker	17	9.4
Housework	8	4.4
**Duration of illness (Month)**		
≥12	31	17.2
13 - 24	33	18.3
25 - 36	32	17.8
37-48	24	13.4
≤49	60	33.3

Analysis of mean and standard deviation of overall sleep quality (M±SD = 11.17 ± 4.69) based on Pittsburgh Sleep Quality Inventory (PSQI) revealed that sleep quality is low in 41.1% of patients with bipolar I disorder, and most of patients suffer from a sleep disorder (Table 2).

**Table 2:** Descriptive statistics of sleep quality (PSQI) in patients with bipolar disorder.

Components (PSQI)	Mean	S.D
Sleep quality	1.37	0.95
Sleep latency	1.83	1.01
Sleep duration	1.32	1.06
Habitual sleep efficiency	1.42	1.02
Sleep disturbances	1.35	0.63
Use of sleeping medications	2.31	1.05
Daytime dysfunction	1.01	0.83
Overall sleep quality	11.17	4.69

The mean and standard deviation of total score of quality of life (M±SD = 56.16 ± 10.81) in bipolar I patients indicated a low QOL in 54.4% of them. Table 3 presents the dimensions of quality of life’s Mean and SD.

**Table 3:** Descriptive statistics of quality of life (WHOQOL-BREF) in patients with bipolar disorder.

Component (WHOQOL-BREF)	Mean	S.D
Physical health	15.12	4.1
Psychological	12.68	3.74
Social relationships	9.21	2.62
Environment	12.55	3.09
The overall quality of life	56.16	10.81

Results revealed that there is a significant relationship between sleep quality and QOL (r=-0.571, *p*<0.001). This relationship is direct, i.e. the QOL diminishes with poor sleep quality (Table 4). In order to find significant predictors of QOL, stepwise regression analysis was run. The result revealed that among seven variables entered as a block, sleep quality and use of sleeping pills were significantly contributed towards the variance of QOL as predictors. These factors accounted for 41% of variance in patients’ quality of life (Table 5).

**Table 4:** Correlation analysis between components of sleep quality and quality of life.

Component of sleep quality	Quality of Life	
	r	*p*-value
Sleep quality	-0.574	< 0.001
Sleep latency	-0.358	< 0.001
Sleep duration	-0.378	< 0.001
Habitual sleep efficiency	-0.382	< 0.001
Sleep disturbances	-0.313	< 0.001
Use of sleeping medications	-0.469	< 0.001
Daytime dysfunction	-0.216	0.004
Overall sleep quality	-0.571	< 0.001

**Table 5:** Stepwise multiple regression analysis of sleep quality on predictive variables (Quality of Life).

Independent variable	Beta	t	R	R^2^	Adjusted R^2^	change R^2^
Sleep quality	-0.574	-9.34	0.574	0.329	0.325	0.329
Sleep quality	-0.467	-7.58				
Use of sleeping medications	-0.306	-4.96	0.641	0.411	0.405	0.082

## DISCUSSION

The results of this study suggested that sleep quality was low and undesirable in 41.1% of patients with bipolar disorder. In many studies, sleep disorders in patients with psychiatric disorders have been mentioned as a major symptom and problem. For example, Hombali et al.^18^ reported the prevalence of sleep disorder in psychiatric patients over 20.75%, which is more common in bipolar disorder (BD); in periods of depression and mania, the incidence of sleep disorder is reported to be over 60%, which differs depending on the study population, definitions, and study methods^19^. Generally, sleep problems in bipolar disorder (BD) are common, but reported rates vary from 10% to 80%, depending on definitions, methodologies and management of potential confounding factors^20^.

Findings of this study corroborate the findings from the study of Gruber et al.^6^, suggesting that insomnia and poor sleep quality has been linked to worse symptom severity and poor outcome in bipolar disorder. Also, Saunders et al.^21^ reported that poor sleep quality at baseline predicted increased severity and frequency of episodes of depression and mania, and frequency of mixed episodes.

The results of study of Sylvia et al.^22^ indicated that sleep disorders are important symptoms of bipolar disorder and should be one of the therapeutic goals to improve sleep quality in patients as there is a significant relationship between sleep disorder and the risk of recurrence of illness. Rocha et al.^23^ reported that BD patients, even in the euthymic phase, had a significantly low sleep quality. Sleep disturbance is associated with a number of poor outcomes including deficits in daytime functioning, increased psychosocial stress, and increases in the utilization of healthcare^24^.

Sleep disturbance in patients with bipolar disorder negatively affected the treatment and care taking of patients as well as their QOL. The study results revealed that the QOL of 54.5% of patients with bipolar I disorder was low and undesirable, which is similar to results reported in other studies^12^^,^^25^^,^^26^.

In similar studies, such as Gomes et al.^27^, it has been reported that the perceived QOL in the BD group is significantly lower than in the healthy subjects. The study of Gutiérrez et al.^28^ also found that patients with BD had a lower QOL, whose health could be improved by treating the symptoms of illness and providing social support. Gazalle et al.^29^ also stated that the symptoms of patients with bipolar disorder were associated with their QOL, so that bipolar patients indicated a lower QOL. Our results also showed that there is a significant relationship between sleep quality and QOL. Sariarslan et al.^30^in their study reported a significant decrease in the life quality of patients with severe sleep problems. Also Palhares et al.^31^stated that bad sleep quality results in decrease the workers’ quality of life.

Overall, sleep disturbances are strongly coupled with inter-episode dysfunction and symptom worsening in bipolar disorder. Research studies suggest that sleep deprivation can trigger manic relapse. There is evidence that sleep deprivation can unpleasantly affect emotion regulation the next day as well as quality of life. The clinical management of the sleep disturbances experienced by bipolar patients, including insomnia, hypersomnia delayed sleep phase and irregular sleep-wake schedule, may include medication approaches, psychological interventions, light therapies and sleep deprivation.

### Limitation

The current study has a number of limitations. The convenience sampling method may limit the generalizability of research outcomes. The cross-sectional design has the disadvantage of only analyzing the research outcomes at a certain point in time. This is a hospital-based sample with subjects who have been treated with medication with a regular follow up. Hence the results cannot be generalized to the community. Future research can focus on above mentioned limitations.

## CONCLUSION

The study suggested that the sleep quality and QOL were low in patients with bipolar I disorder, and there was a statistically significant correlation between these two variables. It can be stated that patients’ sleep quality affects their QOL. Therefore, it is suggested that in the treatment and care plans of these patients, proper planning be done to improve the sleep pattern, which eventually will improve the patients’ QOL. Improving the sleep quality and QOL will help the patient to return to normal life and enhance their personal, occupational and social functions.
